# 207-nm UV Light - A Promising Tool for Safe Low-Cost Reduction of Surgical Site Infections. I: *In Vitro* Studies

**DOI:** 10.1371/journal.pone.0076968

**Published:** 2013-10-16

**Authors:** Manuela Buonanno, Gerhard Randers-Pehrson, Alan W. Bigelow, Sheetal Trivedi, Franklin D. Lowy, Henry M. Spotnitz, Scott M. Hammer, David J. Brenner

**Affiliations:** 1 Center for Radiological Research, Columbia University Medical Center, New York, New York, United States of America; 2 Division of Infectious Diseases, Department of Medicine, Columbia University Medical Center, New York, New York, United States of America; 3 Department of Surgery, Department of Medicine, Columbia University Medical Center, New York, New York, United States of America; MGH, MMS, United States of America

## Abstract

**Background:**

0.5% to 10% of clean surgeries result in surgical-site infections, and attempts to reduce this rate have had limited success. Germicidal UV lamps, with a broad wavelength spectrum from 200 to 400 nm are an effective bactericidal option against drug-resistant and drug-sensitive bacteria, but represent a health hazard to patient and staff. By contrast, because of its limited penetration, ∼200 nm far-UVC light is predicted to be effective in killing bacteria, but without the human health hazards to skin and eyes associated with conventional germicidal UV exposure.

**Aims:**

The aim of this work was to test the biophysically-based hypothesis that ∼200 nm UV light is significantly cytotoxic to bacteria, but minimally cytotoxic or mutagenic to human cells either isolated or within tissues.

**Methods:**

A Kr-Br excimer lamp was used, which produces 207-nm UV light, with a filter to remove higher-wavelength components. Comparisons were made with results from a conventional broad spectrum 254-nm UV germicidal lamp. First, cell inactivation *vs*. UV fluence data were generated for methicillin-resistant *S. aureus* (MRSA) bacteria and also for normal human fibroblasts. Second, yields of the main UV-associated pre-mutagenic DNA lesions (cyclobutane pyrimidine dimers and 6-4 photoproducts) were measured, for both UV radiations incident on 3-D human skin tissue.

**Results:**

We found that 207-nm UV light kills MRSA efficiently but, unlike conventional germicidal UV lamps, produces little cell killing in human cells. In a 3-D human skin model, 207-nm UV light produced almost no pre-mutagenic UV-associated DNA lesions, in contrast to significant yields induced by a conventional germicidal UV lamp.

**Conclusions:**

As predicted based on biophysical considerations, 207-nm light kills bacteria efficiently but does not appear to be significantly cytotoxic or mutagenic to human cells. Used appropriately, 207-nm light may have the potential for safely and inexpensively reducing surgical-site infection rates, including those of drug-resistant origin.

## Introduction

Depending on the procedure, between 0.5% and 10% of all clean surgeries in the US, corresponding to about 275,000 patients per year, result in surgical site infections (SSI) [1]–[3]. Patients who develop SSI are 60% more likely to spend time in an ICU, are 5 times as likely to be readmitted, have a mortality rate twice that of non-infected patients, have an average of 7 days additional length of hospital stay [4], and have roughly double the total healthcare costs compared with patients without SSI [5]. The annual number of deaths in the US attributed to SSI has been estimated at 8,200 [3], with annual patient hospital costs between $3 and $10 billion [6].

A key issue contributing to the extent and severity of the SSI problem is the prevalence of drug-resistant bacteria such as MRSA [7]. One approach which in principle overcomes the problem of drug resistance is the use of germicidal UV lamps [8], [9], because UV light is generally equi-effective at inactivating drug-resistant bacteria compared with wild-type strains [10], [11]. In fact studies of surgical wound irradiation with conventional germicidal UV lamps have shown great promise, with UV fluences corresponding to 4 to 5 logs of MRSA cell kill resulting in significant decreases in SSI rates [8]. However UV radiations in the wavelength range emitted by a germicidal lamp are a human health hazard, causing roughly equal levels of biological damage in human cells as well as bacteria [12]–[14], and are clearly linked with skin cancer [15], [16] and cataract induction [17], [18]. These health hazard to human skin and eyes necessitate the use of cumbersome protective clothing, hoods and eye shields for the surgical staff and the patient [8], [19], and this has prevented widespread use of the UV wound irradiation technique during surgery.

The spectrum of wavelengths emitted by a conventional mercury germicidal UV lamp is broad, covering the UVC, UVB and UVA ranges from about 200 nm to more than 400 nm, with the dominant bactericidal effect due to UVC around 254 nm. Here we suggest and provide evidence here that there exists a narrow wavelength window in the far-UVC region, around 200 nm, in which bacteria are efficiently killed, but which produces far less cytotoxic or mutagenic damage to human cells.

**Figure 1:**
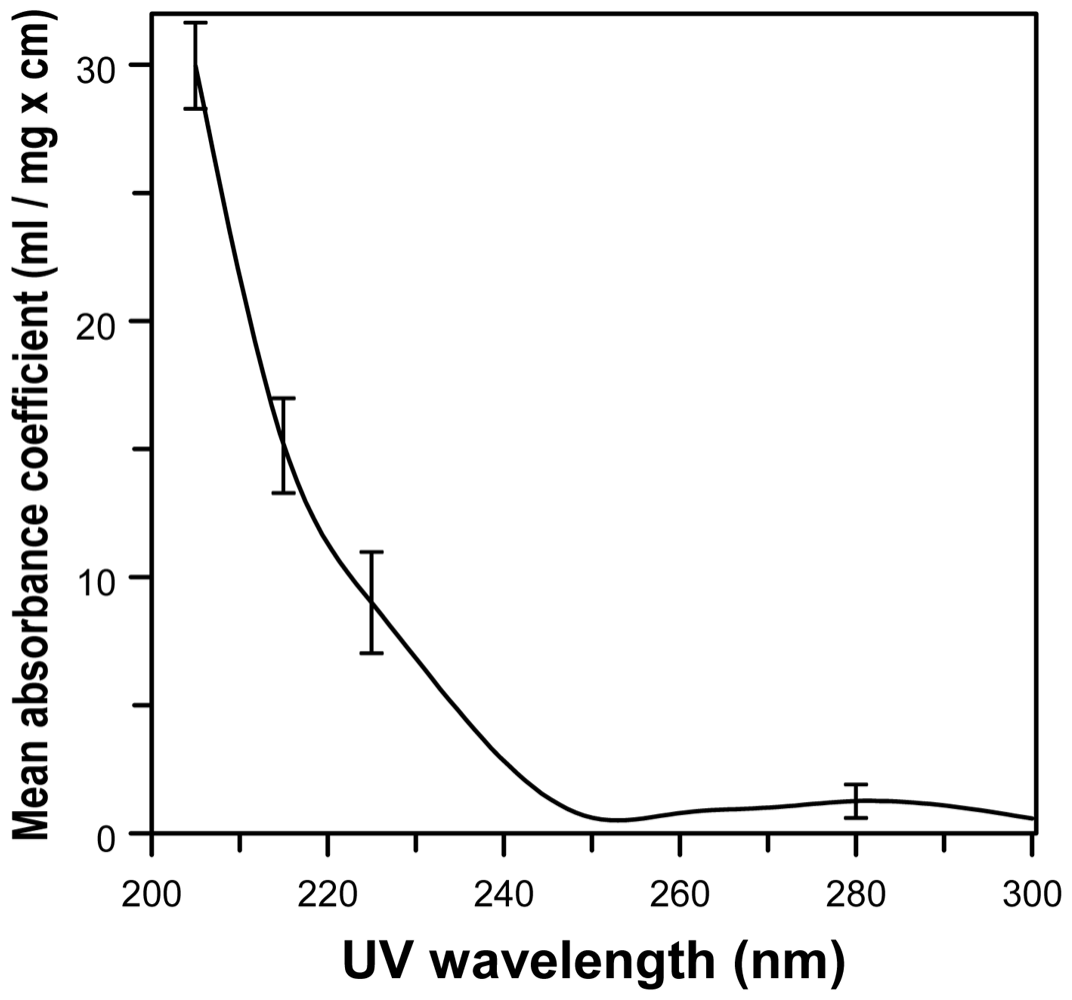
Mean wavelength-dependent UV absorbance coefficients, averaged over published measurements for eight common proteins. Data derived from Ref [24] for human serum albumin, horse hemoglobin, human immunoglobulin-γ, lysozyme, bovine serum albumin, bovine α-chymotrypsinogen, bovine immunoglobulin-γ and bovine ribonuclease A. Typical standard deviations of the measurements across these eight proteins [24] are shown.

The proposed bactericidal application of 207-nm UV light in the presence of humans is based on the fact that UV light at a wavelength of around 200 nm is very strongly absorbed by proteins (particularly through the peptide bond) and other biomolecules [20], [21], so its ability to penetrate biological material is very limited. Thus, for example the intensity of 200-nm UV light is reduced by half in only about 0.3 µm of tissue, compared with about 3 µm at 250 nm and much longer distances for higher UV wavelengths [22], [23]. This phenomenon is quantified in Figure 1 [20], [24]. By contrast, 200-nmUV light is only minimally absorbed in water [25].

As we now discuss, this very limited penetration of ∼200-nm UV light in biological material is significant here, both at the cellular and at the tissue level:

At the cellular level, bacteria are, of course, much smaller than almost any human cell. Typical bacterial cells are less than 1 µm in diameter [26], [27], whereas typical human cells range in diameter from about 10 to 25 µm [26]. It follows that ∼200-nm UV light can penetrate throughout typical bacteria, but cannot penetrate significantly beyond the outer perimeter of the cytoplasm of typical human cells - and will be drastically attenuated before reaching the human cell nucleus. By contrast higher wavelength light from a conventional germicidal lamp can reach human cell nuclei without major attenuation (see Figure 1). Based on these biophysical considerations, while radiation from a conventional UVC lamp would be expected to be cytotoxic and mutagenic to both bacteria and human cells (which is indeed the case [12]–[14]), ∼200 nm UV light would be expected to be cytotoxic to bacteria but very much less cytotoxic or mutagenic to human cells. The current study is designed to test this hypothesis.

At the tissue level, the two organs at major risk from UV light are the skin and the eye [15]–[18]. The key skin cells at risk from UV exposure, such as basal cells and melanocytes, are located within the epidermis below the stratum corneum which is a 5 to 20 µm thick layer of dead non-nucleated cells [28]. Given its very limited penetration (half-value thickness ∼0.3 µm, as discussed above), ∼200-nm light will thus have minimal ability to penetrate the stratum corneum and reach the underlying key skin cells such as basal cells or melanocytes.

Considering now the eye, the most important target from the perspective of UV risk is the lens [17], [18]. The lens is located distal to the cornea, which is sufficiently thick (∼500 µm [29],) such that penetration of ∼200-nm light through the cornea to the lens would be essentially zero [30]. We also need to consider effects on the cornea from the perspective of photokeratitis, though any protective device against eye splash, which is now almost universal amongst surgical staff, would be expected to fully protect the cornea from 207-nm UV exposure.

Overall, these considerations suggest that irradiating the surface of a wound area with ∼200-nm far-UVC light during the course of a surgical procedure may provide all the bactericidal advantages of conventional UV germicidal lamps, while potentially being safe for patient and staff, and requiring no protective clothing. It is the goal of this paper to provide a first test of the hypothesis of the efficacy and safety of ∼200-nm light, using pre-clinical *in-vitro* models of MRSA, human cells and human skin tissue. Studies with animal models are the logical next step.

This approach is made practical by the development [31] of a UV lamp that emits at close to a single wavelength, in this case around 207 nm, defined by the gas mixture it contains; this is in contrast to conventional germicidal mercury UV lamps which emit over a broad range of wavelengths. Such single-wavelength lamps, based on UV emitted from an excited molecule complex (an exciplex), are called excimer lamps, or excilamps [32]. In the wavelength region of interest here, excimer lamps are available containing argon-fluorine, krypton-bromine, or krypton-chlorine mixtures, which respectively produce far-UVC light at 193, 207 and 222 nm [32]. Excimer lamps are small, rugged, inexpensive, sufficiently intense, and long-lived (10,000 to 100,000 h) [32]–[34]; several studies [35]–[38] have been published on the bactericidal properties of far-UVC excimer lamps, but the suggestion that ∼200 nm wavelength UV is differentially cytotoxic to bacteria relative to human cells is new.

Here we use a filtered excimer lamp that produces essentially monoenergetic UV light at 207 nm. We describe measurements of cell killing by 207-nm light, and also by a conventional 254-nm germicidal UV lamp, both for methicillin-resistant *Staphylococcus aureus* (*S. aureus*) cells (MRSA) and also for normal human skin cells. We further describe measurements of typical UV-induced pre-mutagenic DNA lesions in a 3-D human skin model, again both from 207-nm light and from a conventional broad spectrum germicidal UV lamp.

## Materials and Methods

### Production and Characterization of Monoenergetic UV Light at 207 nm

Excimer lamps (excilamps) [31], [32] produce a high-intensity spatially-broad UV beam, primarily at a single-wavelength. The excimer lamp used here was based on a krypton-bromine (Kr-Br) gas mixture, which emits principally at 207 nm. The lamp (High Current Electronics Institute, Tomsk, Russia [32]) was air-cooled and has a 7,500-mm^2^ exit window. For comparison, studies were also carried out with a conventional broad-spectrum low-pressure mercury germicidal lamp (Sankyo Denki G15T8, Japan), with a peak emission at 254 nm.

A UV spectrometer (Photon Control, BC, Canada) sensitive in a wavelength range from 200 nm to 400 nm was used to measure the wavelength spectra emitted by the excimer lamp, and a deuterium lamp standard with a NIST-traceable spectral irradiance (Newport Corp, Stratford, CT, USA) was used to calibrate the UV spectrometer and to estimate absolute photon fluences. A UVX radiometer with a short wavelength sensor (Upland, CA, USA) was used to measure the fluence rate from the conventional germicidal lamp.

**Figure 2:**
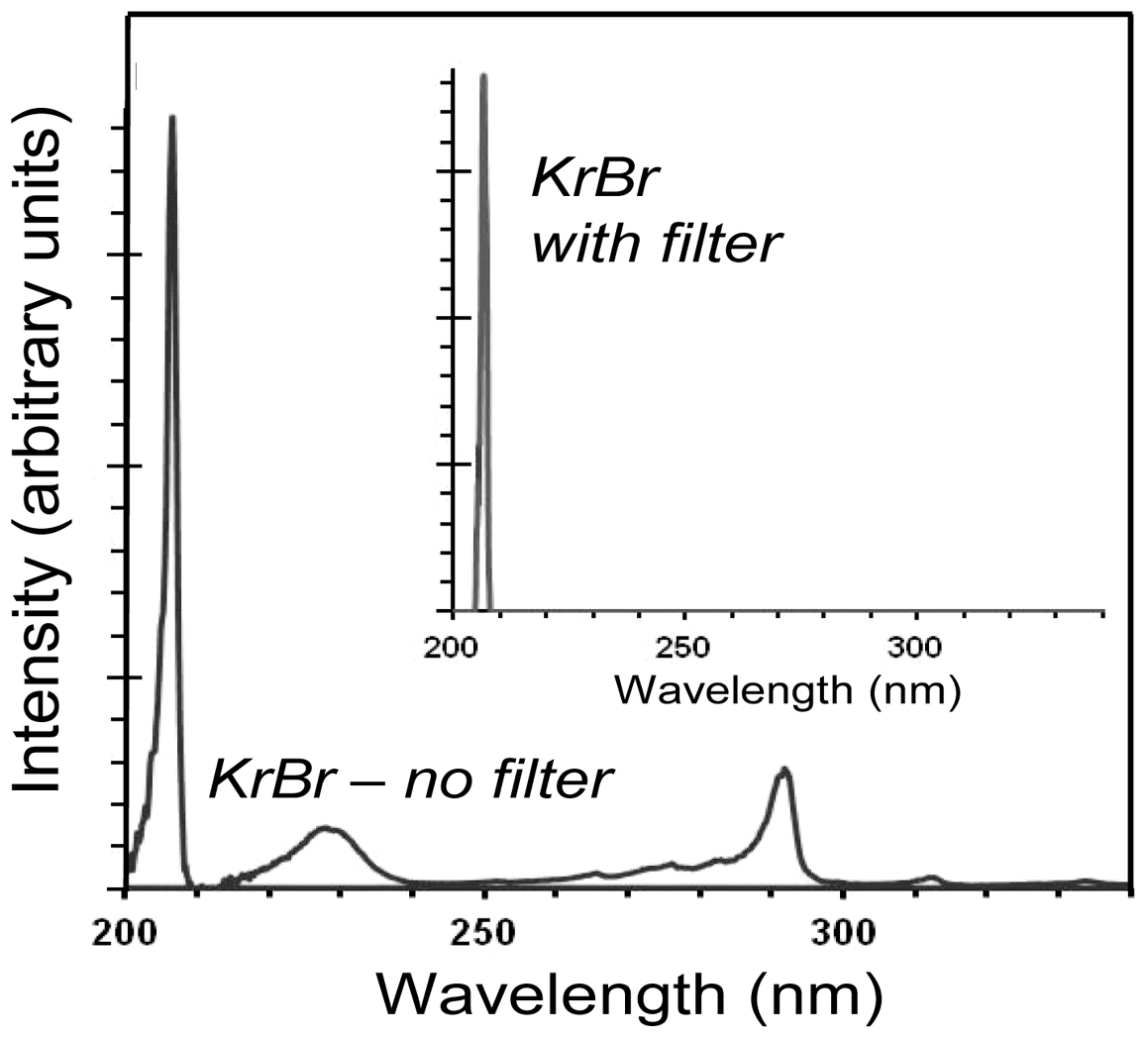
Non-filtered and filtered measured emission spectra from a Kr-Br excimer lamp (main peak 207 nm).

Figure 2 shows measured spectra emitted from the Kr-Br excimer lamp. As well as the main excimer emission at 207 nm, the lamp also emits lower fluences of higher wavelength light, particularly around 228 and 293 nm. These higher-wavelength components are undesirable as they are more penetrating (see Figure 1) and therefore potentially more harmful to human cells. We used a custom bandpass filter (Omega Optical, Brattleboro, VT, USA) to remove all but the dominant 207-nm wavelength emission. Using this filter, the system effectively delivered only a characteristic single wavelength of 207 nm, as shown in the inset to Figure 2.

The UV fluence rates at the target cell location were determined by the lamp intensities and the lamp-target distance. The results shown here used a lamp-target distance of 400 mm, and measured fluence rates at the target from the unfiltered and filtered Kr-Br lamp were respectively 0.33 and 0.044 mW/cm^2^; at the same location, the fluence rate from the conventional UV mercury lamp was 0.45 mW/cm^2^. These fluence rates could be simply adjusted up or down by changing the source-target distance. Depending on the desired fluence, the fluence rates used here resulted in irradiation times from tens of seconds to tens of minutes. Earlier studies reported in the literature showed that, for a given UV fluence, UV inactivation of *S. aureus* is independent of fluence rate/irradiation time over irradiation times from a few seconds to a few hours [39].

### MRSA Cell Survival Assay

Due to their clinical relevance [40], [41], we chose methicillin-resistant *S. aureus* (MRSA) for these initial studies. The strain chosen was USA300 (multilocus sequence type 8, clonal complex 8, staphylococcal cassette chromosome *mec* type IV), originally encountered in the community, but now a major cause of invasive nosocomial infections [42].

Survival of MRSA was assessed using the standard colony forming unit (CFU) assay: Fresh colonies of *S. Aureus* were inoculated into tryptic soy broth (Thermo Scientific, Pittsburgh, PA, USA) and grown overnight at 37°C. The culture was then resuspended in fresh broth and grown to mid-log phase for 3 h. Bacteria were collected by centrifugation, washed, resuspended in broth, and adjusted to an optical density at 600 nm of 0.5. Serial dilutions were performed in HBSS (Hanks’ balanced salt solution), to yield initial bacterial densities from 10^8^ to 10^−1^ CFU/ml, depending on the planned UV fluence. 50 µl of the desired dilution was then spread onto standard 100-mm diameter prepared media plates (containing a substrate of tryptic soy agar with 5% sheep blood; BD Diagnostic System, Sparks, MD, USA) using a glass spreader, resulting in a bacterial monolayer on top of the substrate. Almost all the HBSS used in the spreading was rapidly absorbed into the agar substrate underneath the cells; we measured the average depth of the residual HBSS covering the bacterial monolayer at the time of irradiation, which was <0.01 mm. We confirmed that this residual HBSS would not significantly attenuate the 207 nm UV light, through direct transmission measurements of 207 nm UV through the HBSS, which was >99.9% after 2 mm of HBSS, and therefore >>99.9% after 0.01 mm of HBSS.

Three media plates were used for each fluence point, and the plates were irradiated from above for different time intervals, depending on the required fluence. The plates were then incubated overnight at 37°C, with CFU colony counting performed the following day. Bacterial inactivation was expressed as the proportional reduction in CFU/mm^2^, relative to sham irradiated zero-fluence controls.

### Normal Human Cell Survival Assay

Normal human diploid skin fibroblasts (AG1522) were obtained from the American Type Culture Collection (Manassas, VA, USA). Cells at passage 10–12 were grown in Eagle’s Minimum Essential Medium (CellGro, Manassas, VA, USA) supplemented with 12.5% heat-inactivated (56°C, 30 min) fetal calf serum, 200 mM L-alanyl-L-glutamine, 100 U/ml penicillin and 100 µg/ml streptomycin (Sigma-Aldrich Corp., St. Louis, MO, USA). The cells were maintained at 37°C in a humidified incubator with 5% CO_2_ in air. Cells were seeded in glass chambers (Nalge-Nunc Lab-Tek, Rochester, NY, USA) at a density to reach confluence about 48 h before exposure. Under these conditions, 90–98% of the cells were in G_0_/G_1_ phase of the cell cycle, as determined by tritiated-thymidine incorporation and flow cytometry.

Cell survival was determined by the standard colony formation assay [43]. Briefly, cells were trypsinized within 5–10 min of exposure, suspended in growth medium, counted, diluted and seeded in 100-mm dishes with numbers resulting in ∼100 clonogenic cells per dish. Three replicates were done for each data point. After an incubation period of 12 to 14 days, the cells were rinsed with phosphate buffered saline (PBS), fixed in 95% ethanol and stained with crystal violet. Macroscopic colonies with more than 50 cells were scored as survivors. Measured survival values were corrected for the system plating efficiency which ranged from 20% to 30% as measured with sham-irradiated controls.

### Measurement of UV-associated Pre-mutagenic DNA Lesions in 3-D Human Skin Model

The 3-D human skin model used was a full skin thickness construct which recapitulates all the key components of human skin, within the epidermal (including the stratum corneum) and the dermal layers [44]. The construct (EpiDerm-FT, purchased from MatTek Corp., Ashland, MA, USA) consists of organized basal, spinous, granular, and cornified layers analogous to those found *in vivo*.

The two pre-mutagenic DNA lesions assayed in the epidermis were cyclobutane pyrimidine dimers (CPDs) and pyrimidine-pyrimidone 6-4 photoproducts (6-4PPs), which are generally considered the major pre-mutagenic DNA lesions linked with UV-induced skin cancer [16].

The two lesions were assayed and scored using standard immunohistochemical approaches, following the protocols described, for example, by Stolper et al [45] in Epiderm-FT constructs and by Qin et al [46]. In brief, after UV irradiation, the EpiDerm-FT cultures were fixed in 10% neutral buffered formalin (overnight, room temperature), paraffin embedded, sectioned with a microtome, and stained with hematoxylin and eosin. 5 µm paraffin-embedded cross sections mounted on glass slides were deparaffinized and heat-mediated antigen retrieval was conducted. Slides were incubated in PBS followed by 0.05 N HCl at 4°C for 5 min before being washed sequentially in PBS, 50% EtOH and 70% EtOH, 0.15 N NaOH, 70% EtOH, 50% EtOH, and PBS for 2 min each at room temperature. Slides were blocked in 1% bovine serum albumin in PBS for 1 h followed by mouse anti-CPDs (Kamiya Biomedical Company, Seattle, WA, USA) or mouse anti-6-4PPs (Cosmo Bioscience USA, Carlsbad, CA, USA) 1∶50 in blocking buffer for 1 h at room temperature. Samples were washed in PBS (3 times, 5 min each) and incubated for 1 h with a biotinylated secondary antibody (1∶200 in PBS) followed by a 5 min wash in PBS, streptavidin-HRP (Sigma-Aldrich Corp., St. Louis, MO, USA) for 30 min, and finally DAB substrate-chromogen solution for 15 min as specified by EMD Millipore protocol (Billerica, MA, USA). The samples were then washed with water and covered with methyl green counterstain solution at room temperature for 3 min. The samples were dipped in 100% EtOH twice and into xylene. Finally, cover slips were mounted on each sample with mounting medium (Vectashield, Burlingame, CA, USA). CPD- and 6-4PP positive cells were scored in a total of nine randomly chosen locations in the epidermis from three different tissue samples.

## Results

### 1 Cell Survival

**Figure 3:**
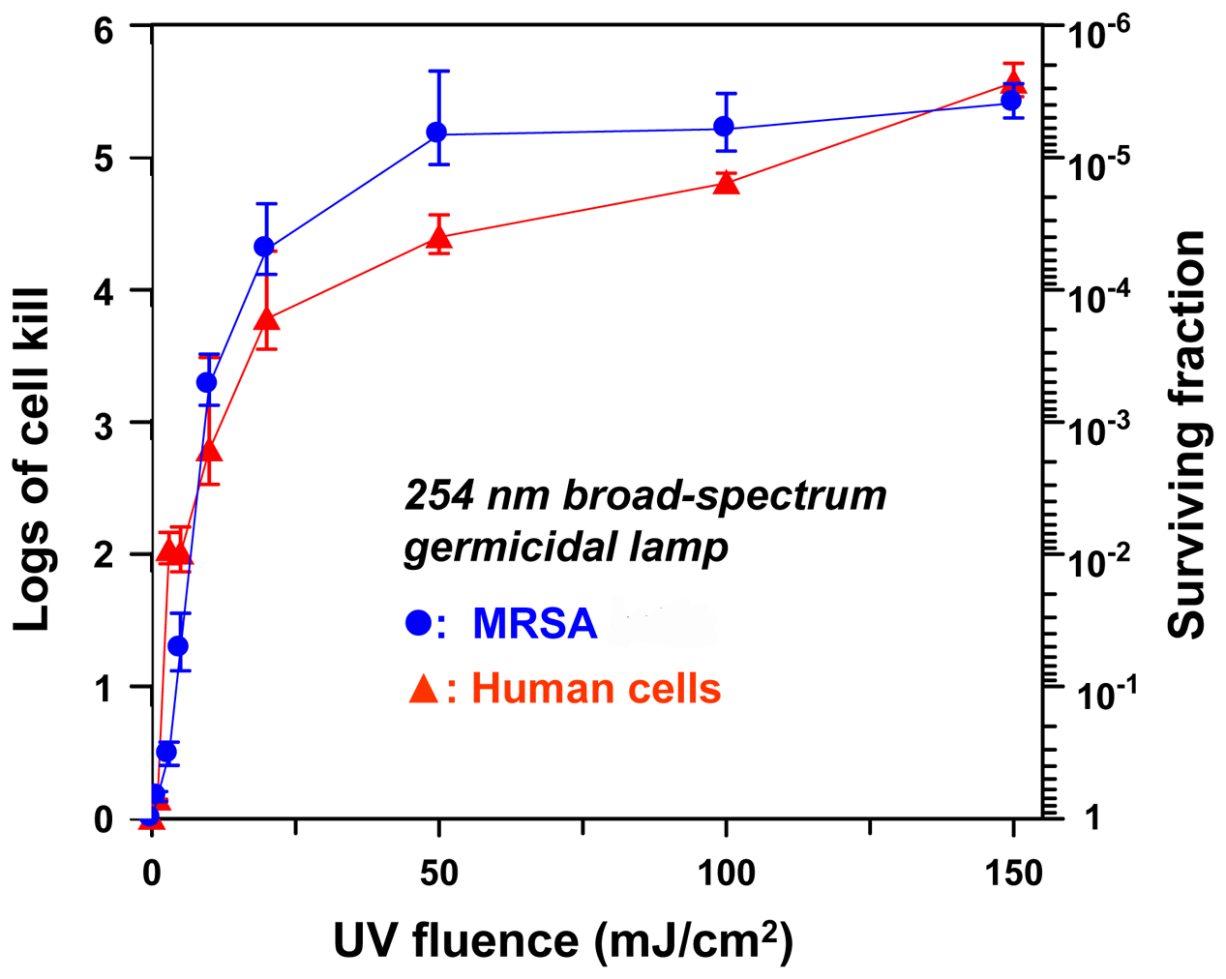
Cell killing of MRSA and human cells, induced by UV from a conventional germicidal lamp. Cell killing is shown relative to zero-fluence controls, expressed as surviving fraction, or as logs of cell kill (-log_10_[surviving fraction]), produced by different fluences of UV exposure from a conventional broad-spectrum germicidal UV lamp (peak emission 254 nm). Data are shown for methicillin resistant *S. aureus* cells (•, MRSA strain USA300) and for AG1522 normal human fibroblasts (▴).

**Figure 4:**
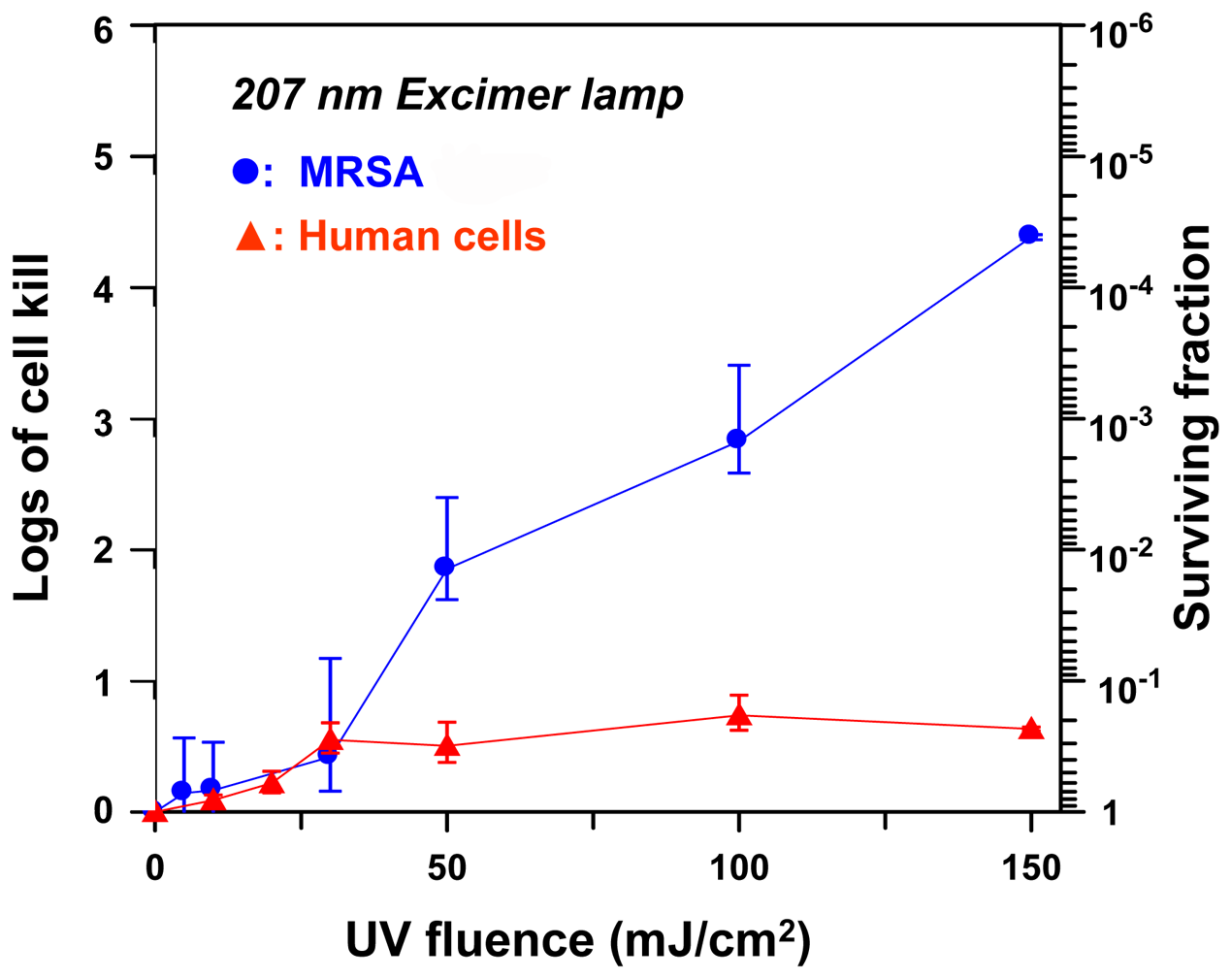
Cell killing of MRSA and human cells, induced by 207-nm UV light. Cell killing is shown relative to zero-fluence controls, expressed as surviving fraction or as logs of cell kill (-log_10_[surviving fraction]), produced by different fluences of UV light from a filtered 207-nm Kr-Br excimer lamp. Data are shown for methicillin resistant *S. aureus* cells (•, MRSA strain USA300) and for AG1522 normal human fibroblasts (▴).

We measured cell survival in MRSA (strain USA300) and in normal human skin fibroblasts (AG1522) exposed to different fluences of UV light generated by a 254-nm broad-spectrum germicidal UV lamp (Figure 3), and by 207-nm UV light from a filtered Kr-Br excimer lamp (Figure 4).

As illustrated in Figure 3, the conventional broad-spectrum germicidal UV lamp (broad spectrum, peak 254 nm) efficiently kills MRSA cells but, as expected, is almost equally efficient at killing human cells. By contrast, as illustrated in Figure 4, at the fluences studied here, the 207-nm excimer lamp provided up to four logs of MRSA cell killing but produced little cell killing in human cells – quantitatively about a 5,000 fold differential in cell killing between MRSA and human cells.

For example, four logs of MRSA cell killing (cell survival level of 10^−4^) required fluences of 35 mJ/cm^2^ of 254-nm (broad-spectrum) UV and 135 mJ/cm^2^ of 207-nm light; these fluences respectively produced cell killing in human cells of 2×10^−4^ (254 nm) and 2×10^−1^ (207 nm). Thus for the same level of MRSA killing, the 207-nm excilamp produces about 1,000-fold less killing in human cells compared to a conventional germicidal lamp.

This major differential in human cell killing between broad-spectrum 254 nm UV and 207 nm light is consistent with that observed by Kochevar *et al.* [47], [48], who compared the effects of broad spectrum 254-nm UV with 193-nm laser light; consistent with the hypothesis in the current work, they attributed the low level of human cell killing by 193-nm laser light to non-DNA based membrane damage.

### 2 Induction of Pre-mutagenic DNA Lesions in Human Skin

**Figure 5:**
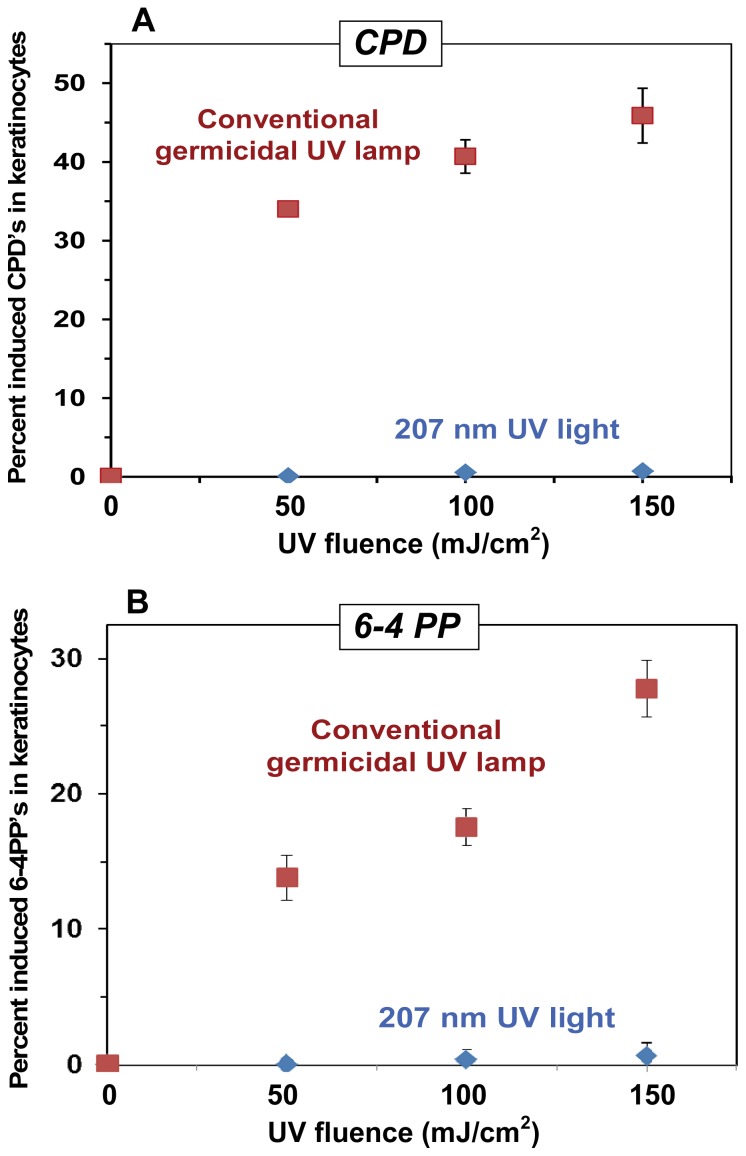
Pre-mutagenic skin DNA lesion yields induced by a conventional germicidal lamp and by 207-nm UV light. Yields are shown of pre-mutagenic DNA lesions in epidermal keratinocytes, measured in a 3-D human skin tissue model, induced by conventional broad-spectrum germicidal UV radiation (▪) and by 207-nm UV light (♦). **A:** cyclobutane pyrimidine dimers (CPD); **B:** pyrimidine-pyrimidone 6-4 photoproducts (6-4PP). In both graphs zero-fluence control measurements (<1%) have been subtracted from the data.

Figure 5 shows the measured induced yields of CPD lesions (Figure 5a) and 6-4 photoproducts (6-4PP, Figure 5b) after exposure of the 3-D skin tissue model to the broad UV spectrum from a conventional germicidal UV lamp (peak 254 nm) or to 207-nm light from the Kr-Br excimer lamp. As expected, the germicidal lamp produced high yields of both pre-mutagenic skin DNA lesions. However after 207-nm exposure, neither lesion showed an induced yield which was significantly elevated above zero, at any of the studied fluences.

## Discussion

The results shown here are in accord with biophysical expectations based on the limited penetration of 207-nm UV light: in terms of cell killing, 207-nm light does indeed kill MRSA much more efficiently than it kills human cells. For example at a fluence of 207-nm light that produce four logs of MRSA cell kill, there is much less than one decade of cell kill in human cells (as discussed above, we chose four logs of bacterial kill for this comparison because studies of wound irradiation with conventional UV germicidal lamps suggest that this level of bacterial reduction will produce significant decreases in SSI rates [8]). In contrast with the results for 207-nm light, while conventional germicidal lamps efficiently kill MRSA, conventional germicidal lamps are almost as efficient at killing human cells. Quantitatively, for a four-log level of MRSA killing, 207-nm UV light produces about 1,000-fold less human cell killing than a conventional germicidal UVC lamp.

In terms of the safety of 207-nm UV light, the lack of induction of typical UV-associated pre-mutagenic DNA lesions in the epidermis of a 3-D skin model is again consistent with biophysical expectations based on the limited penetration of 207-nm UV light, and consistent with earlier studies using 193-nm laser light [23]. Whilst other UV wavelengths have been suggested as potentially safe for human exposure, in particular 405 nm [49] and 254 nm [50], no mechanisms have been proposed for a differential toxic effect for bacteria *vs*. human cells at these wavelengths, and indeed both 405-nm light [51] and 254-nm light [52] have been shown to be mutagenic to mammalian cells at relevant fluences.

Based on the pre-clinical *in-vitro* studies reported here, 207-nm light from a Kr-Br excimer lamp may have considerable potential for safely reducing SSI rates. Such lamps could potentially be used in an operating room environment without the need for protective clothing for staff or patient. Of course *in-vivo* studies are a necessary confirmation and these are ongoing, as discussed below.

Should *in-vivo* studies confirm these *in-vitro* results, our expectation of the potential use of 207-nm light would be for continuous low-fluence-rate exposure during a surgical procedure. The rationale here is that current evidence suggests that the majority of SSI result from bacteria alighting directly onto the surgical wound from the air. Evidence for the dominance of an airborne route comes from correlations between the density of airborne bacteria and postoperative sepsis rates [53], [54]; evidence for the significance of airborne bacteria alighting directly on the surgical wound comes, for example, from studies of conventional UV lamps specifically directed over the surgical site [8], and also wound-directed filtered airflow studies [55].

Thus a continuous low-fluence-rate exposure of 207-nm UV light onto the surgical wound area during the complete surgical procedure might be anticipated to kill bacteria as they alighted onto the wound area. Such a continuous exposure, as also used in the earlier studies of wound irradiation with conventional germicidal lamps [8], would be designed to inactivate bacteria before they penetrated into the interior of the wound. A second advantage associated with targeting bacteria as they alight onto the wound area is in relation to biofilms [56]: as bacteria alight onto the skin/wound, they are typically in individual planktonic form, and thus amenable to killing by 207-nm light. This would prevent the subsequent formation of bacterial clusters (biofilms), which would probably be refractory to 207-nm light (as they are to most other therapies).

Several configurations for the 207-nm lamp in a surgical setting might be possible. It is of course important that surgical staff do not inadvertently block the UV light, so one arrangement might be for the excimer lamp to be incorporated into a standard overhead surgical illumination system. A possible second UV light source, to ensure a level of redundancy from inadvertent shielding, might be incorporation into a surgeon’s headlight illumination system, with the UV light transmitted to the headlight via fiber optics [57].

Commercial 207-nm excimer lamps would be expected to be inexpensive and long lived. In fact in a different wavelength range (172 nm) xenon excimer lamps [58] with rated lifetimes of 50,000 to 100,000 hrs, are already in commercial use [34], providing (using appropriate phosphors) both interior and exterior office lighting, for example on the Rotterdam KPN Telecom tower [59].

Though beyond the scope of the current work, in addition to direct bactericidal applications in surgical environments, it might be envisioned that 207-nm UV light could be used to treat, on a continuous basis, any airborne environment where there is a high likelihood of airborne-based pathogen transmission, such as TB or pandemic influenza. In fact upper-room UV irradiation systems have long been considered, based on conventional broad-spectrum UV lamps [60], and have shown some promise [61], [62]; they have not, however, been widely used, in part because of safety concerns relating to potential low-level broad-spectrum UV exposure [61], [63], [64].

## Conclusions

There is clearly no single solution to the major problem of SSI [65]. Conventional germicidal UV lamps are effective but represent a health risk both for patient and staff. Basic biophysics considerations suggest, and our *in-vitro* data provide some confirmation that, 207-nm UV light has a considerably improved therapeutic ratio (bacterial killing *vs*. cell human killing) compared to conventional germicidal UVC lamps [50], and also that 207-nm UV light is unlikely to be mutagenic to humans. Based on these considerations, 207-nm light has considerable promise to be safe and inexpensive modality for SSI reduction, while potentially sharing with conventional UV germicidal lamps [8] the major advantage of being equally effective for drug-resistant and drug-sensitive bacteria.

Of course before 207-nm light approach were to be used clinically in a surgical scenario, a number of issues remain to be addressed: *In vivo*, studies of safety and efficacy in animal models of SSI are ongoing, including the potential effects of UVC exposure on wound healing - though earlier studies [66] have not shown significant effect on wound healing.
